# The role of complement C5a receptor in DPSC odontoblastic differentiation and in vivo reparative dentin formation

**DOI:** 10.1038/s41368-022-00158-4

**Published:** 2022-01-27

**Authors:** Muhammad Irfan, Ji-Hyun Kim, Hassan Marzban, David A. Reed, Anne George, Lyndon F. Cooper, Seung Chung

**Affiliations:** 1 Department of Oral Biology, University of Illinois at Chicago, Chicago, USA; 2 Department of Human Anatomy and Cell Science, University of Manitoba, Winnipeg, Canada

**Keywords:** Stem-cell differentiation, Stem-cell research

## Abstract

Therapeutic dentin regeneration remains difficult to achieve, and a majority of the attention has been given to anabolic strategies to promote dentinogenesis directly, whereas, the available literature is insufficient to understand the role of inflammation and inflammatory complement system on dentinogenesis. The aim of this study is to determine the role of complement C5a receptor (C5aR) in regulating dental pulp stem cells (DPSCs) differentiation and in vivo dentin regeneration. Human DPSCs were subjected to odontogenic differentiation in osteogenic media treated with the C5aR agonist and C5aR antagonist. In vivo dentin formation was evaluated using the dentin injury/pulp-capping model of the C5a-deficient and wild-type mice. In vitro results demonstrate that C5aR inhibition caused a substantial reduction in odontogenic DPSCs differentiation markers such as DMP-1 and DSPP, while the C5aR activation increased these key odontogenic genes compared to control. A reparative dentin formation using the C5a-deficient mice shows that dentin regeneration is significantly reduced in the C5a-deficient mice. These data suggest a positive role of C5aR in the odontogenic DPSCs differentiation and tertiary/reparative dentin formation. This study addresses a novel regulatory pathway and a therapeutic approach for improving the efficiency of dentin regeneration in affected teeth.

## Introduction

Dental caries is one of the major health issues which affects the majority of the U.S. adult population.^1^ The severity of the carious injury determines the dentin-pulp complex response. For example, in case of a moderate injury, the surviving odontoblasts produce protective reactionary dentin beneath the injured site,^2,3^ while a serious injury may involve full or partial regeneration including vascularization, innervation, and dentin repairment endorsed by the generation of odontoblast-like cells.^4^ It causes severe pain if left untreated and requires endodontic therapy or may lead to permanent tooth loss.^5^ There could be several culprits behind caries; including bacterial invasion, proteolysis or physicochemical dissolution of teeth components, and direct interaction of bacteria or their toxins with dental pulp stem cells (DPSCs) and odontoblasts trigger a reparative process of tertiary dentin formation which further recruit and differentiate DPSCs.^6^

In clinical therapies, pulp-capping could be aimed to repair dentin and sustain its vitality which ultimately leads to the endurance of natural dentition.^7^ However, post infection, therapeutic dentin regeneration is unclear and the underlying mechanism staging the role of inflammation on dentinogenesis in dentin-pulp regeneration is to be elucidated.

The complement system, a key player of innate immunity and inflammation, is expressed and activated in the carious teeth.^8^ Beyond its role in immunity, the complement system participates in tissue regeneration of the liver,^9^ bone^10^, and cardiac tissues.^11^ C5a is the complement component active fragment activated from plasma proteins in response to injury. Since C5a is a powerful chemotactic factor, it is involved in one of the early steps in dentin-pulp regeneration by recruitment of the immune cells and human pulp progenitor cells to the injured area.^12–14^ It exerts its action by binding to the G-coupled protein receptor (GPCR) C5aR.^15,16^ Previously, we also demonstrated that complement C5aR activation is involved in two major processes of dentin-pulp regeneration: the pulp progenitor’s migration to the injured site^17,18^ and the pulp nerve growth beneath carious injury.^19–21^

Very little information is available about the involvement of the complement in the tooth’s response to the common infection, caries. Moreover, inflammatory complement C5a’s role in caries-mediated dentin regeneration has received little recognition. We recently identified the role of C5aR and C5L2 in DPSCs odontoblastic differentiation under hypoxia and inflammatory context using C5aR antagonist and C5L2 siRNA.^22,23^ Here, we further propose a significant role for the complement system and C5aR in DPSCs odontoblastic differentiation by activating C5aR and in vivo reparative dentin formation using the C5a-deficient mice.

## Results

### Differentiating DPSCs express mesenchymal stem cell marker STRO-1 and C5a receptor

**Fig. 1:**
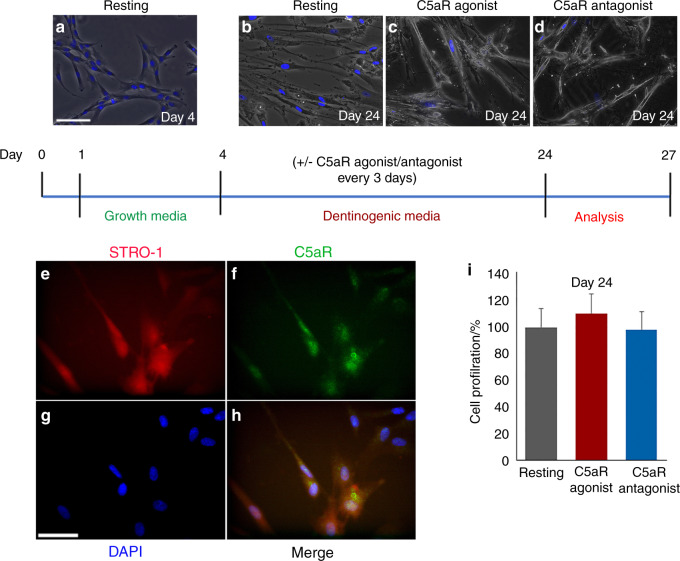
A schematic timeline representation of odontogenic DPSCs differentiation. Commercially available DPSCs were acquired and confluent DPSCs were cultured in a regular growth media for 4 days. 2–4 passage of DPSCs were cultured at 5 × 10^4^ cells per well concentration. Subsequently, cells were cultured in dentinogenic media and treated with a C5aR agonist or antagonist from the differentiation day 4 to day 24. The medium was replaced every 3 days. Representative images of differentiating DPSCs at day 4 (**a**) and day 24 (**b**–**d**). **e**–**h** Double-immunofluorescence staining with anti-STRO-1 (red channel) and C5aR (green channel) in DPSCs at day 4 confirms that C5a receptor-positive cells also express STRO-1. **i** Bar graph showing no. of differentiated cells as cell proliferation. Differentiated DAPI stained cells were counted using ImageJ software. Scale bars: 100 μm (**a**–**d**), and 50 μm (**e**–**h**)

Human DPSCs were subjected to odontogenic differentiation using the osteogenic medium for 24 days. We have successfully set up in vitro DPSCs odontoblastic differentiation before.^22,23^ Figure 1b–d shows DPSCs differentiated into odontoblast-like cells at day 24 compared to differentiating DPSCs at day 4 (Fig. 1a) as previously described by Baldion et al.^24^ and Huang et al.^25^. The C5a receptor agonist and inhibitor W54011 were applied during differentiation at day 4 (a timeline in Fig. 1). The homogeneous population of DPSCs was examined with the co-localization of the mesenchymal stem cell marker STRO-1 (Fig. 1e). Our analysis confirms over 99% purity of DPSCs (*P* < 0.001). These cells have been validated in several recent publications.^22,23,26^ Previous studies revealed that human DPSCs express the C5aR.^18^ Consistent with these data, our immunofluorescent double staining data indicated that the STRO-1-positive DPSCs express the C5aR (Fig. 1e–h). No significant difference in the number of the cell population was observed among various treatments (Fig. 1i).

### C5a receptor activation resulted in a significant increase in DSPP and DMP-1 expression in the differentiated cells from DPSCs

**Fig. 2:**
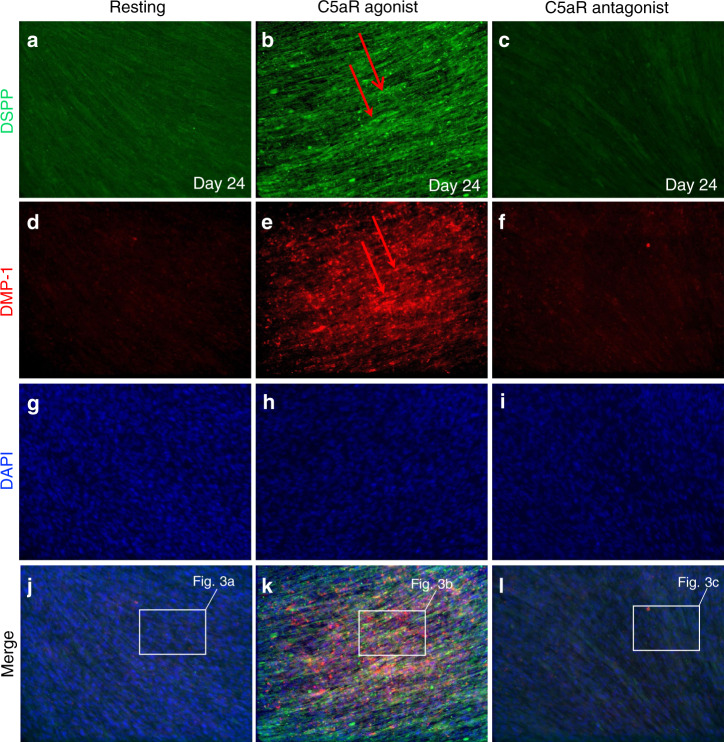
Complement C5a receptor-mediated DSPP and DMP-1 expression after DPSCs odontoblastic differentiation. Immunofluorescence double staining was used to analyze DSPP and DMP-1 expression in C5aR-mediated DPSC differentiation. Anti-DSPP (green) and DMP-1 (red) were found co-localized in cytoplasm of DPSCs in C5aR-agonist-treated cells after 21 days of treatment (**b**, **e**, and **k**). An intense staining was observed in C5aR-agonist-treated cells. For each condition, negative controls, performed by replacement of the DSPP and DMP-1 primary antibodies and secondary antibody used to detect DSPP was Alexa-488 (green; **a**-**c**) and DMP-1 through Alexa-594 (red; **d**−**f**); nuclei were counterstained with DAPI (blue; **g**−**i**). (**j**−**l**) Merged figures showing colocalization of DSPP and DMP-1 among resting, C5aR-agonist and C5aR-antagonist treated DPSCs.

To identify the role of the complement receptor C5aR activation and blocking in the DPSCs odontoblastic differentiation, DPSCs were cultured and subjected to odontogenic differentiation for 3 weeks using dentinogenic media (Fig. 2). Cells were treated with complement C5a receptor (C5aR) agonist or antagonist every three days.

**Fig. 3:**
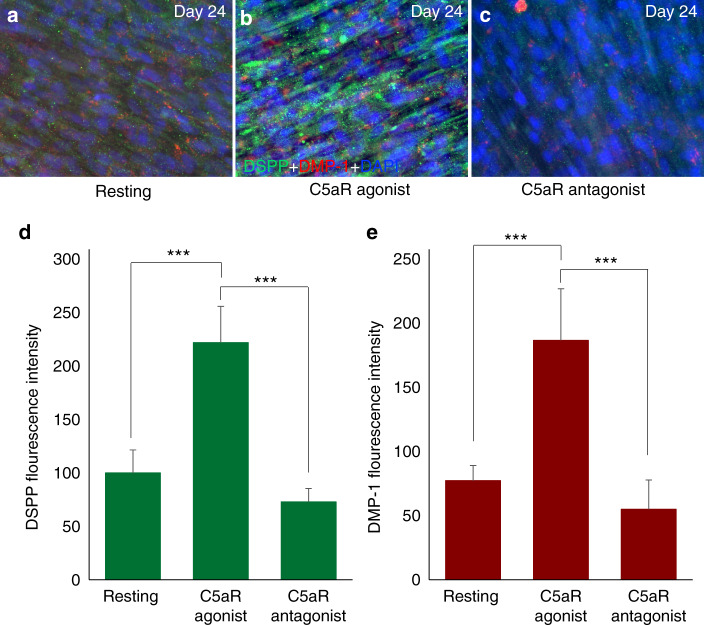
DPSC-derived odontoblastic cells express more DSPP and DMP-1 under C5aR agonist treatment. **a**–**c** Magnified pictures (**a**–**c**) from the respective boxes of Fig. 2. Anti-DSPP (green) and DMP-1 (red) immune intensities are significantly higher in the C5aR agonist treatment group (**b**) compared to resting (**a**) and C5aR antagonist treatment (**c**) groups. **d**, **e** Bar graphs showing the fluorescence intensity of DSPP and DMP-1 in various treatment groups. C5aR stimulation by C5aR agonist specifically increases DSPP (**d**) and DMP-1 (**e**) expression in DPSCs undergoing odontoblastic differentiation (gray versus red lines). ***P < 0.001 vs untreated control of resting cells (*n* = 3).

The differentiated cells showed odontoblast-like properties as they expressed well-established odontogenic cell markers^27,28^ such as dentin matrix protein (DMP)-1 and dentin sialophosphoprotein (DSPP). The treatment of DPSCs with C5aR agonist significantly increased the expression of DSPP and DMP-1 (Fig. 2b, e). Immunofluorescence staining shows that C5aR-agonist-stimulated DPSCs expressed significantly increased DSPP, and DMP-1 expression compared to resting cells, after 21 days of differentiation (Fig. 2j, k). Higher magnification shows the co-localization of DSPP and DMP-1 in the cytoplasm of DPSCs in C5aR-agonist treatment groups (Fig. 3b). Staining density of DSPP and DMP-1 was observed significantly higher (Fig. 3d, e; *p* < 0.001) in C5aR-agonist-treated cells (221.8 ± 33.7 and 186.6 ± 40.2, respectively) compared with resting control (100 ± 21.4 and 77.2 ± 11.7). Taken together, these data suggest that C5aR activation plays a key role in the modulation of DSPP and DMP-1 secretion, linking these key molecules to the mechanisms of action of C5aR.

### C5a receptor blocking resulted in a decrease in DSPP and DMP-1 expression in the differentiated cells from DPSCs

Moderate inhibition of DSPP and DMP-1 expression was observed in C5aR-antagonist-treated cells (Figs. 2c, f, l and 3c), indicating the involvement of complement C5aR in the expression of important proteins that help mineralization and development of hard dentin. The bar graph shows the fluorescence intensity between various treatment groups (Fig. 3d, e). Staining density of DSPP and DMP-1 significantly reduced (Fig. 3c; *P* < 0.001) in C5aR-antagonist-treated cells (72.8 ± 12.5 and 54.8 ± 22.9, respectively) compared with the agonist-treated group (221.8 ± 33.7 and 186.6 ± 40.2, respectively), and it was even less than resting control cells. These results suggest the involvement of complement C5a as a positive regulator in dentinogenesis.

### In vitro stimulation of DPSCs with recombinant human C5a has no effect on the expression of DSPP

**Fig. 4:**
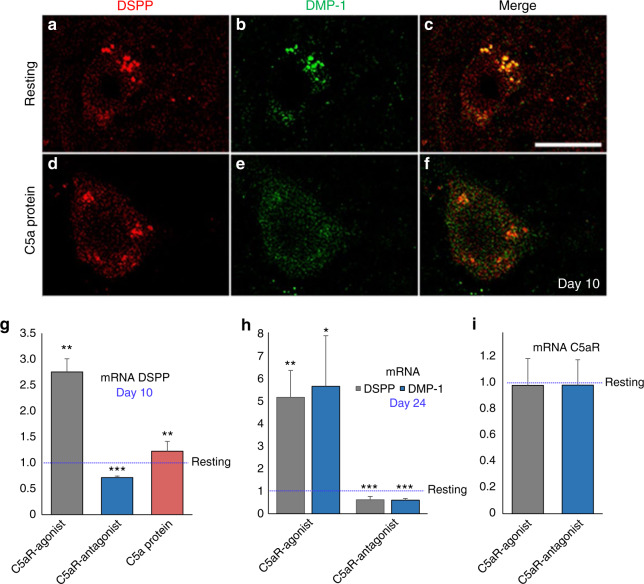
Effect of exogenous C5a on DSPP and DMP-1 expression and PCR analysis of DPSC-derived odontoblast-like cells. **a**–**f** Immunofluorescence double staining was used to analyze DSPP and DMP-1 expression in exogenous C5a-mediated DPSCs at day 10. Anti-DSPP (red) and DMP-1 (green) were found weakly co-localized in the cytoplasm of DPSCs in C5a-treated cells without any significant difference compared with resting control. Scale bar: 25 μm. **g**, **h** Real-time PCR analysis of DSPP and DMP-1 in the C5aR agonist, C5aR antagonist, and C5a recombinant protein treatment groups compared to control (calculated ‘1’ value of respective genes expression). The analysis reveals a significant upregulation of DSPP and DMP-1 mRNA expression in the C5aR agonist treatment group. Treatment of DPSCs using the recombinant human C5a protein does not affect the basal level of DSPP expression. **i** C5aR mRNA expression between resting cells and C5aR-agonist or antagonist-treated cells. C5aR-agonist or antagonist treatment do not induce a C5aR mRNA expression change

Our previous studies showed that recombinant C5a protein had no effect on the pulp fibroblast-mediated brain-derived nerve growth factor^19^ and nerve growth factor secretion,^21^ we next determined whether exogenous C5a treatment influences the DSPP expression on DPSCs. Double-immunofluorescence-labeled staining for anti-DSPP and anti-DMP-1 during early differentiation shows that DSPP and DMP-1 were weakly co-localized in the cytoplasm of DPSCs in C5a-treated cells (Fig. 4a–f). Real-time PCR analysis of DSPP mRNA expression at day 10 by DPSCs demonstrates early and continued induction during odontoblastic differentiation (Fig. 4g). Treatment of DPSCs using the recombinant human C5a protein does not affect the basal level of DSPP expression but significantly increased with C5aR-agonist treatment (Fig. 4g—C5aR agonist: 2.78 ± 0.24 vs C5aR antagonist: 0.68 ± 0.049 vs C5a protein: 1.16 ± 0.21; *P* < 0.01; *P* < 0.001). Similarly, C5aR-agonist treatment significantly increased the mRNA levels of DSPP and DMP-1 at day 24 (Fig. 4h; *P* < 0.05; *P* < 0.01) while C5aR-antagonist inhibited them (Fig. 4h; *P* < 0.001), without affecting C5aR expression level (Fig. 4i).

### C5a deficiency reduces the regenerative dentin formation of injured teeth

**Fig. 5:**
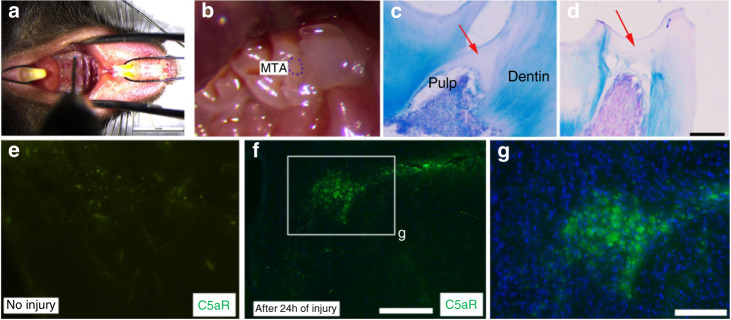
C5aR expression increased in injured teeth of wild-type mice. **a**, **b** 0.3 mm drill tip was used to puncture the dentin until the upper surface of the pulp, and shortly after, the injured area was capped with MTA. Scale bar: 5 mm. **c**, **d** Hematoxylin and eosin staining showed a reparative dentin formation in the control mice (red arrows) after 4 weeks of recovery. Scale bar: 250 μm. **e**–**g** Immunofluorescence labeling for C5aR (green) reveals that after 24 h of the injury, C5aR expression is significantly increased in the injured area (**f**; Scale bar: 250 μm, **g**; Scale bar: 50 μm.) compared to control (**e**)

To identify in vivo role of C5a in reparative dentin formation, we utilized wild-type and C5a-deficient mice. The targeted disruption of the C5a gene was described previously.^29^ To induce dentin injury to wild-type (WT) mice (Fig. 5), the burr exposed the dentine/pulp, and a hypodermic needle was used to further punch the pulp. The exposed dentin/pulp was sealed with mineral trioxide aggregate (MTA) to protect the pulp from further inflammation (Fig. 5b). Animals were sacrificed after 4 weeks of the initial injury, and the mandibles were removed. Histological and immunofluorescence staining was performed to evaluate the importance of complement system activation in dental pulp response to carious injuries. After 24 h of the injury, C5aR expression is significantly increased in dental pulp (Fig. 5f, g) compared to control (Fig. 5e).

**Fig. 6:**
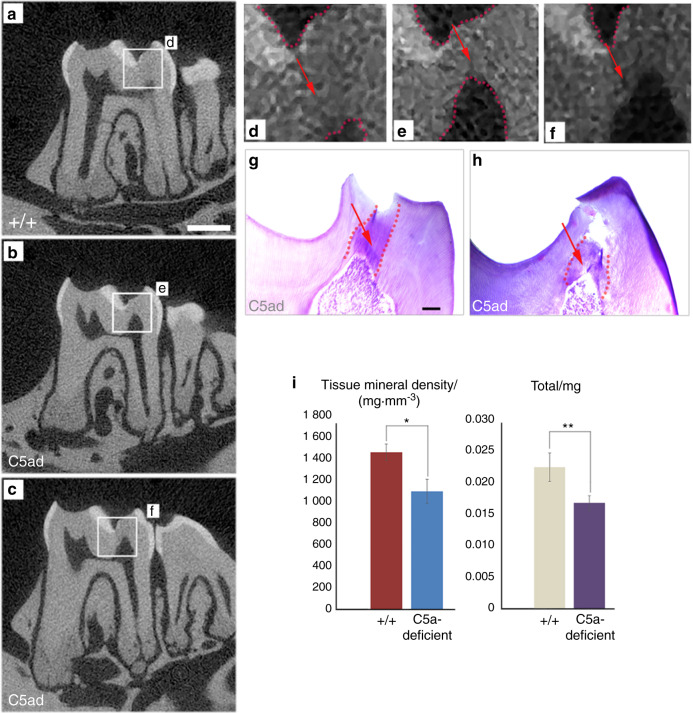
In vivo dentin regeneration in the C5a-deficient mice after dentin injury/pulp-capping. **a**–**c** Representative Micro-CT images of upper molars obtained from C5a-deficient (C5ad) mice (**b**, **c**) and respective control (**a**) after 4 weeks of dentin injury. Scale bar: 0.5 mm. **d**–**f** The rectangle area of **a**, **b**, **c** is magnified in **d**, **e**, and **f**. Reparative dentin (red arrows) was observed after 4 weeks of recovery. C5a-deficient mice show a decreased dentin regeneration compared to the wild-type controls. **g**–**h** H&E staining of C5a-deficient (C5ad) mice shows delayed and defective dentin formation. **i** Quantitative calculation of dentin regeneration in the C5a-deficient mice. Tissue mineral density and Total mg of regenerated volume is statistically analyzed. Mineral formation analysis at the regeneration area shows that C5a-deficient mice form less mineral than the control. *P < 0.05, **P < 0.01. Scale bar: 100 μm

The reparative/tertiary dentin formation in the teeth of WT mice was evaluated through the light microscopy observation of H&E-stained sections (Fig. 5c, d). The serial sections of the whole teeth were sectioned with a thickness of 15 μm and stained and collected to reveal its overall pattern. Also, a micro-CT analysis was performed to quantitatively measure the regenerated dentin volume and mineral density. Representative micro-CT images (Fig. 6a–f) and H&E-stained sections (Fig. 6g, h) of C5a-deficient mice teeth and quantitative calculations (Fig. 6i) of dentin regeneration show less mineralization in the C5a-deficient mice after 4 weeks of recovery. The decrease is statistically significant—Tissue Mineral Density (TMD): C5a^+/+^: 1453 ± 78 vs C5a^−/−^: 1093 ± 111.3; Total mg: C5a^+/+^: 0.022 ± 0.002 vs C5a^−/−^: 0.016 ± 0.001. Taken together, our comprehensive analyses demonstrate that C5a deficiency resulted in a decreased dentin regeneration suggesting the C5a’s key in vivo role in reparative dentin formation.

## Discussion

Inflammation, which is a natural host response to infection/injury, is known to control dentinogenesis both positively and negatively. It facilitates the pulp progenitor cell’s migration, proliferation, and odontoblastic differentiation.^8,17,18^ However, uncontrolled inflammation can cause irreversible damages to the tissue. Clearly, bacteria and their toxins influence odontoblasts during advanced caries affecting dentin.^30^ New odontoblast-like cells could help in dentin repair for subsequent regeneration,^12,31^ but this complex regenerative process is mainly associated with DPSCs and their key factors involved in activation and differentiation.^8^ Literature has suggested that dentin extracellular matrix molecules or some growth factors *i.e*., transforming growth factor beta-1 or basic fibroblast growth factor which produced because of acid dissolution of dentin, are responsible for dentin regeneration following caries or dentin infection.^18,19,32,33^ However, it remains unclear how regenerative signals are linked to inflammation and trauma.

We and others recently identified complement system activation, a central and early response to tissue damage, as an additional source of regeneration signals for dentin regeneration subsequent to carious injuries.^8,19^ One of the most critical components of innate immunity and inflammation is the complement system which can be activated by multiple factors like apoptosis, necrosis, or some pathogen-associated molecular patterns.^34–36^ Such events could be observed during caries. Carious injuries trigger the complement system by cytolytic membrane attack complex formation in human third molars.^17,18^ This is considered among the most effective initial responses against pathogens and altered host cells^37^ including the recruitment of immune cells due to the production of important anaphylatoxin C5a and some opsonins like C5b.^38^ Beyond its role in immunity, the complement system participates in tissue regeneration of the liver,^9^ bone^10^, and cardiac tissues.^11^ Recently, our lab has established that complement system activation in two critical steps of dentin regeneration: recruitment of pulp progenitor cells to the site of injury^17,18^ and the pulp nerve growth beneath carious injury.^19–21^ However, there are a lack of studies that implicate the complement activation system in the odontoblastic differentiation of DPSCs and tertiary dentin formation or regeneration in response to caries. Here we have explored this phenomenon in cell culture and animal models, and we demonstrate that C5aRs expression is elevated in inflamed pulp tissue.

Our findings supporting a key role for the complement C5a receptor in DPSC odonto-lineage differentiation and in vivo dentin regeneration are supported by a recent study where Bergmann et al.,^39^ explained the linkage between inflammation and dental tissue regeneration through complement activation. Previous studies have explored the differentiating potential of DPSCs into odontoblast-like cells by confirming their genotypic expressions and phenotypic characteristics using DSPP and DMP-1 immunofluorescence staining.^24,25^ Similar attributes were observed in our study (Figs. 1 and 2). DSPP and DMP-1 are expressed in differentiating odontoblasts and are essential proteins for dentin formation by enhancing mineralization.^40^ Further, the differentiated DPSC’s showed odontoblast-like phenotype as they expressed well-established odontogenic cell markers DMP-1 and DSPP (Figs. 2 and 3), yet at reduced levels in the presence of W54011, indicating the direct involvement of complement C5a proteins. Matsui et al.^41^ has revealed the role of DSPP and DMP-1 in CD146-positive DPSC mediated dentin-pulp regeneration. Dentin regeneration is a complex process involving many biological pathways. For example, Volponi et al.^42^ has reported the Wnt signaling pathway and its importance for odontoblastic differentiation and reparative dentin formation. Their group observed that OCN-Cre;Wls^fl/fl^ transgenic mice exhibited stronger DSPP expression in odontoblasts and pulp, and denser dentin formation^43^ due to reduced Wnt signaling. Another published study showed that DSPP might be a downstream signaling effector to the DMP-1 in dentinogenesis using transgenic mice.^44^ D’Souza et al.^45^ has evaluated the in vivo developmental functions of DSPP and DMP-1 and concluded that DSPP and DMP-1 play a distinct role in the dentin matrix and their expression is evident throughout the process of odontogenesis. Our results show that treatment of DPSC with C5aR agonist significantly increased the DSPP and DMP-1 expression (Figs. 2 and 3). This is the first study showing that C5aR activation in DPSCs could lead to a significant amount of dentin formation. This observation was consistent with our in vivo dentin regeneration in wild-type mice (Fig. 5) and the study using C5a-deficient mice (Fig. 6).

Previously, we designed a modified and choreographed microenvironment and established that dentin-pulp regeneration is a locally regulated process.^4,8^ Botero et al.^46^ has identified odontoblasts as a source of regeneration signals in caries but deep injuries may lead to the disintegration of odontoblasts at the injured area.^12^ Chmilewsky et al.^22^ has stated that C5a stimulation did not affect the expression of DMP-1 in DPSCs. Similarly, we did not find any effect of recombinant C5a on DSPP expression. Previously, our group has also reported that human pulp cells interact with the recombinant C5a^18^ and also demonstrated that human pulp progenitor cells bind the C5a generated by the complement system activation from pulp fibroblast-produced proteins after LPS stimulation.^47^ To explore the role of C5a and to see whether the inflammation could affect the complement activation and at injured pulp, we studied C5a-deficient mice. Here, we observed that the complement system is activated in mouse teeth under the influence of carious injury which is directly proportional to the severity of injury while the inflammation was also weakly triggered in the C5a-deficient mice lacking complement system activation. It is well-known fact that there is a strong linkage between dental tissue regeneration and inflammation.^39^ Thus, the reduced inflammation in C5a-deficient mice might have decreased both odontogenesis and tertiary/reparative dentin formation (Figs. 5 and 6). In our unpublished observation, we have confirmed that LPS-induced inflammation potentiates C5aR-mediated dentinogenesis and this is p38 protein kinase-dependent. Taken together, our results confirmed that C5aR has a positive regulatory role in odontogenic DPSCs differentiation and reparative dentin formation.

It has long been established that DPSCs have a high regeneration capacity. Dentin regeneration is the key factor for maintaining tooth preservation following infection or injury. We believe that still there is a lot to explore about the involvement of complement system activation and its relation to bone and tissue regeneration. Understanding the biological mechanisms of odontogenesis/dentinogenesis and the role of inflammation in this process are required for successful DPSC engineering strategies. The scientific knowledge obtained from this study provides a foundation for creating therapeutic tools that target DPSC during dentin-pulp complex regeneration.

## Materials and methods

### Chemicals and reagents

Human dental pulp stem cells (DPSCs) were purchased from Lonza, Pharma & Biotech (Cat. # PT-5025). C5aR antagonist – W54011 was acquired from MilliporeSigma (Cat. # 234415; Billerica, MA, USA) and C5aR agonist from Anaspec (Cat. # AS-65121; Fremont, CA, USA). MEM-alpha, PBS, fetal bovine serum, L-glutamine, and Antibiotic-Antimycotic were procured from Gibco™ Fisher Scientific (Waltham, MA, USA). Poly-D-Lysine coated (BioCoat™, 12 mm) round German glass coverslips slips were purchased from Corning™ Fisher Scientific (Cat. # 354087; Waltham, MA, USA). Various antibodies were procured: anti-C5a receptor (Cat. # 21316-1-AP; Proteintech, ST. Louis, MO, USA), mouse anti-DMP-1 (Cat. # SAB14002752; R&D System/Sigma, ST. Louis, MO, USA), mouse anti-STRO-1 (Cat. # sc-47733; Santa Cruz, Dallas, Texas, USA), mouse anti-DSPP (Cat. # sc-73632; Santa Cruz, Dallas, Texas, USA).

### Animals

Male C57BL/6, C5a-deficient mice aged 6-7 weeks and weighing 20–22 g were purchased from Jackson laboratory (Cat. #000461). The mice were acclimatized for 1 week in an air-conditioned room with a 12 h/12 h light/dark cycle at a temperature and humidity of 22 °C ± 2 °C and 55% ± 10%, respectively, before further experimentation.

### Cell culture

Commercially available human DPSCs, which were guaranteed through 10 population doublings, to express CD105, CD166, CD29, CD90, and CD73, and to not express CD34, CD45, and CD133; were further evaluated by immunocytochemistry in cultures with the STRO-1, a stem cell marker. Our analysis confirms that over 99% of cells (*n* = 5) used for our study were DPSCs. DPSCs were cultured at 37 °C and 5% CO_2_ in regular/osteogenic media and treated with C5aR antagonist – W54011 (10 nmol·L^**−**1^) or C5aR agonist (20 nmol·L^**−**1^) for 72 h in regular growth media (α MEM containing 10% fetal bovine serum (FBS), 1% L-glutamine and antimycotic/antibiotic), and then swapped with osteogenic media (α MEM containing 20% FBS, 1% l-glutamine and antimycotic/antibiotic, supplemented with 100 μg·mL^**−**1^ ascorbic acid, 10 mmol·L^**−**1^ β-glycerophosphate and 10 mmol·L^**−**1^ dexamethasone) for 21 days and treated every three days with agonist or antagonist. The medium was changed every 2–3 days. The whole differentiation experiments were conducted with different sets of DPSCs (between 2nd and 4th passages) 3 times, and cell proliferation was measured by counting the total number of cells.

### Real-time PCR

DPSCs were cultured according to the differentiation protocol mentioned above in 6 wells plate with triplicates at 5 × 10^4^ cells per well concentration for the treatment of the C5a receptor agonist or inhibitor W54011. Total mRNA was extracted with 0.8 mL Trizol (Invitrogen, Waltham, MA, USA) and analyzed using the Fisher Scientific NanoDrop 2000 device. The cDNA samples were analyzed using the Applied Biosystems SYBR green reagent system according to the manufacture protocol. Primer sequence of DSPP; forward: 5′-CTG TTG GGA AGA GCC AAG ATA AG-3′, reverse: 5′-CCA AGA TCA TTC CAT GTT GTC CT-3′.

### Immunohistochemistry

Mice teeth were fixed and routinely processed as previously described.^14^ Paraffin-embedded tissues were sectioned from intact and carious teeth and deparaffinized with xylene and graded ethanol. Some sections were hematoxylin-eosin-stained. For others, an antigen retrieval was performed at 98 °C for 20 min in Tris 1 mmol·L^**−**1^ per 0.1 mmol·L^**−**1^ EDTA/0.5% Tween, pH 6.0 and nonspecific binding sites were blocked with 0.25% casein in PBS for 15 min at room temperature. Then tooth sections were probed with primary antibodies with mouse anti-human C5aR (20 μg·mL^**−**1^) or their respective isotype control for 1 h at room temperature and secondary antibody for 45 min with Alexa Fluor-488 rabbit anti-mouse IgG (2 μg·mL^**−**1^), and DAPI (1 μg·mL^**−**1^) counterstain for fluorescence microscopy. Diaminobenzidine (DAB, 0.5 mg·mL^**−**1^) was used to visualize the reaction.

### Immunofluorescence staining

Differentiating and differentiated DPSCs were fixed and permeabilized as previously described.^19,21^ Subsequently, cells were incubated overnight with rabbit anti-C5a receptor (1:200), mouse anti-DMP-1 (1:1 000), mouse anti-STRO-1 (1:200), mouse anti-DSPP (1:500) or their respective control/isotypes. Later, the cells were treated with secondary antibody for 3 h with a mix of Alexa Fluor-594 anti-mouse IgG, Alexa Fluor-488 anti-rabbit IgG (1 μg·mL^−1^), and/or DAPI (2 μg·mL^**−**1^). The coverslips were mounted, and images were taken using a Zeiss Axiovert microscope. Fluorescence density was quantified using ImageJ 1.49v software and values were analyzed for statistical significance by SAS 9.4.

### In vivo dentin regeneration

Male C57BL/6 C5a-deficient mice were purchased from Jackson laboratory (#000461). Mice homozygous for the targeted mutation are viable, fertile, normal in size, and do not display any gross physical or behavioral abnormalities. A 0.3 mm rounded carbide burr drill with an automatic speed was applied to penetrate the dentin of the mouse upper first molar (only left side, the right side was used as a control). Once the burr exposes the dentine/pulp, an 11 G needle was used to further punch the pulp. The exposed dentin/pulp was sealed with mineral trioxide aggregate (MTA) to protect the pulp from further inflammation. Animals were sacrificed starting after 4 weeks of the initial injury and the mandibles removed.^48–51^ The molars were examined and scored for smooth surface caries.

### Data analysis

The statistical analysis was performed on at least 3 independent experiments with duplicates or triplicates, and statistical significance was determined using the student’s *t*-test (SAS 9.4) to compare the different treatments and their respective controls (*P*-value < 0.05 or less was considered statistically significant). For quantification of immunofluorescence staining intensity, ImageJ 1.49v software was used. Fixed areas of 1 mm × 1 mm or 2 mm × 2 mm were selected to analyze the number or fluorescence intensity of differentiated cells. Micro-CT analyses were performed using the RUSH hospital’s commercial service to quantitatively measure the regenerated dentin volume. All animal phenotype analyses were blinded by examiners to avoid the examiner’s preferences or expectations.
